# The regulatory effects of realgar and cinnabar on glucose metabolism in mice

**DOI:** 10.3389/fendo.2025.1658148

**Published:** 2025-11-24

**Authors:** Yifan Zhang, Qingsong Qu, Ertong Dai, Ruiyan Liu, Bilin Jin, Zixuan Lu, Hui Kong, Yue Zhang, Huihua Qu, Yan Zhao

**Affiliations:** 1 School of Traditional Chinese Medicine, Beijing University of Chinese Medicine, Beijing, China; 2 School of Life Sciences, Beijing University of Chinese Medicine, Beijing, China; 3 Center of Scientific Experiment, Beijing University of Chinese Medicine, Beijing, China

**Keywords:** realgar, cinnabar, diabetes, glycometabolism, enzymatic activity

## Abstract

**Background:**

Glucose metabolism plays a central role in maintaining systemic energy homeostasis, and its dysregulation is closely linked to the pathogenesis of metabolic diseases such as diabetes mellitus. While traditional mineral medicines such as realgar and cinnabar have a long history of use, their roles in glycometabolism remain poorly defined.

**Methods:**

Male C57BL/6J mice were used to establish both normoglycemic and streptozotocin (STZ)-induced diabetic models. Oral glucose, starch, protein, fat, and cellulose load tests were performed to evaluate the effects of realgar and cinnabar on postprandial glycemia and thermoregulation. Random blood glucose, body temperature, and insulin levels were monitored. α-Glucosidase and α-amylase inhibition assays were conducted *in vitro* to explore potential digestive enzyme-targeted mechanisms.

**Results:**

Realgar and cinnabar significantly reduced blood glucose levels in diabetic mice and attenuated postprandial glycemic excursions in normal mice following oral glucose and starch loading. Further analysis revealed elevated insulin levels and dose-dependent inhibition of α-glucosidase and α-amylase activities. However, these hypoglycemic effects were abolished when glucose was administered intraperitoneally, and no significant changes in blood glucose were observed under non-carbohydrate nutrient loads (fat, protein, or fiber).

**Conclusion:**

This study provides the first systematic evidence that realgar and cinnabar exert hypoglycemic effects, which involving enhanced insulin secretion and inhibition of key digestive enzymes. Their substrate-specific actions and partial influence on thermoregulation suggest broader roles in metabolic regulation and warrant further investigation in chronic models and energy homeostasis pathways.

## Graphical Abstract

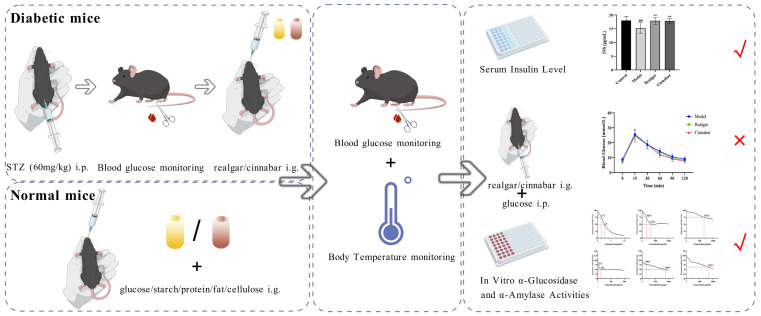

## 1 Introduction

Glucose metabolism plays a central role in maintaining physiological homeostasis and serves as one of the primary sources of cellular energy. The regulation of blood glucose levels depends on a complex interplay of hormonal signals and enzymatic activities across multiple organs, including the liver, pancreas, skeletal muscle, and adipose tissue. This coordinated system manages energy intake, storage, and utilization (1). When this tightly regulated network is disrupted, it can lead to a variety of metabolic disorders, most notably diabetes mellitus. In recent years, the incidence of glycometabolism disorders has steadily increased, driven by changes in dietary patterns and the rising intake of refined carbohydrates, the global diabetic population is projected to climb to 853 million by 2050, becoming a significant public health burden (2, 3). Beyond diabetes, impaired glucose metabolism has been shown to be closely associated with the development of cardiovascular diseases, neurodegenerative disorders, polycystic ovary syndrome, and certain cancers (4, 5). As such, developing precision-targeting therapies for glucose metabolism intervention holds significant clinical and societal value.

It is important to emphasize that disturbances in glucose metabolism are not only reflected in abnormal blood glucose levels but are often accompanied by a broader imbalance in the overall energy metabolism system. Glucose metabolism is not only a critical process for ATP production but also regulates thermogenesis, redox status, and multiple signaling pathways, playing a key role in the coupling of fat and protein metabolism (6). When glucose availability is limited, the body typically activates compensatory mechanisms such as fatty acid β-oxidation or protein catabolism to meet basic energy demands (7). However, prolonged metabolic reprogramming can lead to mitochondrial dysfunction, increased oxidative stress, and reduced metabolic flexibility, accelerating the development of metabolic syndrome (8, 9).

In this context, the role of metal elements in glucose metabolism has gradually been elucidated, providing potential research directions for the intervention of metabolic disorders. Recent studies have shown that deficiencies or imbalances of metal elements may be significant risk factors for diabetes and its related complications. Deficiencies in elements such as calcium, chromium, iron, and magnesium have been linked to metabolic disturbances, including insulin resistance and prediabetes (10, 11). Supplementation of these metal elements has demonstrated potential in improving insulin resistance and regulating blood glucose levels. Notably, the role of metal elements in the treatment of diabetes has garnered widespread attention in recent years.

Traditional mineral medicines containing metal elements, such as Realgar (As_4_S_4_) and Cinnabar (HgS), have long been documented in classical Chinese pharmacopeias including the Ben-Cao-Gang-Mu and the Shen-Nong-Ben-Cao-Jing, where they are described as tranquilizing the mind and inducing sedation. Modern pharmacological investigations have partially validated traditional perspectives, demonstrating that realgar and cinnabar possess significant regulatory effects on the central nervous system. These include anxiolytic, anticonvulsant, and sedative activities, as reported in recent studies (12, 13). In parallel, the anticancer potential of Realgar has garnered increasing attention. A growing body of evidence suggests that Realgar can suppress the proliferation of various tumor cell lines through mechanisms involving mitochondrial apoptosis and reactive oxygen species (ROS)-mediated pathways (14–16). Furthermore, nano-formulated Realgar has demonstrated enhanced antitumor efficacy in preclinical models of hepatocellular carcinoma and glioblastoma (17–19). Notably, during our preliminary pharmacological screening, we serendipitously observed that both Realgar and Cinnabar exhibited potential corrective effects on glucose metabolism disorders. However, to date, no studies have systematically investigated the roles or mechanisms of these mineral medicines in glycometabolic regulation. Thus, their potential utility in the treatment of metabolic diseases remains largely unexplored. To further substantiate these findings, the present study employed streptozotocin (STZ)-induced diabetic mouse models alongside healthy mouse to systematically investigate the regulatory effects of Realgar and Cinnabar on glucose metabolism and the activity of key digestive enzymes. The aim is to provide theoretical support and experimental evidence for the potential modern application of mineral-based traditional medicines in the treatment of metabolic diseases.

## 2 Materials and methods

### 2.1 Chemicals and reagents

Realgar and cinnabar were purchased from Hebei Hehuachi Pharmaceutical Co., Ltd. (Hebei, China). Streptozotocin (STZ, S0130) was purchased from Sigma-Aldrich Corporation (St. Louis, MO, USA). Sodium citrate buffer solution (C1013) was purchased from Beijing Solarbio Technology Co., Ltd. (Beijing, China). Sodium carboxymethyl cellulose (C104985) and soluble starch (S104452) were purchased from Shanghai Aladdin Bio-Chem Technology Co., Ltd. (Shanghai, China); α-glucosidase (S10050), α-amylase (S31302), PNPG (S10137), DNS (R27125), acarbose (S11190), and glucose (A10014) were obtained from Shanghai Yuanye Biotechnology Co., Ltd. (Shanghai, China). Peptone (FP410) was purchased from Angel Yeast Co., Ltd. (Hubei, China). Cellulose (X0401) was purchased from Hefei BASF Biotechnology Co., Ltd. (Anhui, China). Lard was purchased from Beijing Wumart Supermarket (Beijing, China). Blood glucose test strips were obtained from Guilin Zhonghui Technology Development Co., Ltd. (Guilin, China). Insulin assay kits were purchased from Cloud-Clone Corp Co., Ltd. (Wuhan, China).

### 2.2 Animals

Specific-pathogen-free (SPF) C57BL/6J male mice (body weight 24 ± 2 g) were purchased from Beijing Vital River Laboratory Animal Technology Co., Ltd (Beijing, China; certificate number: SYXK (Beijing) 2023-0011). Upon arrival, animals were allowed to acclimate for seven days in an accredited animal facility before the onset of any experimental procedures. During this adaptation period, mice were housed in ventilated cages with corncob bedding under controlled environmental conditions (temperature 23 ± 2°C, relative humidity 55 ± 5%, and a 12-hour light/12-hour dark cycle). Sterilized pelleted chow and autoclaved water were provided ad libitum. All husbandry and experimental protocols were carried out in strict accordance with the regulations of Beijing University of Chinese Medicine on the management and use of experimental animals (Approval No. BUCM-2025022101-1053). All animal experiments also complied with the internationally recognized Guide for the Care and Use of Laboratory Animals (8th edition, National Academies Press, 2011) (20). The study design followed the principles of the 3Rs (Replacement, Reduction, and Refinement) to ensure ethical use of animals, with group sizes selected to achieve scientific objectives while minimizing animal use.

### 2.3 Model establishment and drug administration

Operational Procedure for Glucose Metabolism in Normal Mice: Twenty-four male mice were acclimated and then randomly assigned to three groups (n=8 each): Control, Realgar (15 mg/kg body weight (mg/kg bw)), and Cinnabar (75.8 mg/kg bw). After a 12-hour fast with free access to water, tail-tip blood was collected at 0, 15, 30, 60, 90, and 120 minutes to measure glucose levels, and body temperature was recorded at 0, 30, 60, and 120 minutes. Mice in each group received their designated test substance by oral gavage in numerical order; the Control group was given an equivalent volume of 0.9% saline. Immediately after gavage, all animals were administered 20% glucose solution by oral gavage in the same sequence. Per the experimental protocol, metabolic changes in starch (1600 mg/kg bw), protein (1600 mg/kg bw), fat (1600 mg/kg bw), and cellulose (1600 mg/kg bw) were assessed in batches using the same procedures. Rate of blood glucose change (%) = |baseline glucose level - glucose level after observation period|/baseline glucose level × 100%.

Establishing the Diabetic Model and Dosing: Forty male mice were randomly assigned to four groups (n=10 per group): Control, Model, Realgar, and Cinnabar. Following a 12-hour fast with free access to water, diabetes was induced by intraperitoneal injection of streptozotocin (STZ; 60 mg/kg bw, dissolved in 1% sodium citrate buffer) for three consecutive days. Seventy-two hours after the final STZ injection, tail‐tip blood was collected to measure blood glucose; random blood glucose levels exceeding 13.8 mmol/L were considered indicative of successful diabetic modeling (21). Upon confirmation of hyperglycemia, mice in each group received daily oral gavage as follows: Blank and Model groups were given 0.5% carboxymethylcellulose sodium (CMC-Na) vehicle, Realgar group (15 mg/kg bw), and Cinnabar group (75.8 mg/kg bw). Doses for realgar and cinnabar were calculated based on the maximum daily dosage specified in the Chinese Pharmacopoeia and were suspended in 0.5% CMC-Na.

### 2.4 Enzyme-linked immunosorbent assay

After two weeks of treatment, blood was collected from diabetic mice, allowed to clot at 4°C for 6 h, and centrifuged at 3,000 rpm for 10 min at 4°C to obtain serum. Serum insulin concentrations were then measured using an ELISA kit according to the manufacturer’s instructions.

### 2.5 α-Glucosidase inhibition assay

α-Glucosidase activity was measured using p-nitrophenyl-α-D-glucopyranoside (PNPG) as substrate. In each 2 mL centrifuge tube, 40 µL of α-glucosidase (2 U/mL) was mixed with 40 µL of test sample at five concentrations (100, 10, 1, 0.1, and 0.01 mg/mL). Tubes were pre-incubated at 37°C for 10 min, then 40 µL of 10 mmol/L PNPG in 0.1 M phosphate buffer (pH 6.8) was added. After a further 30 min incubation at 37°C, the reaction was stopped by adding 80 µL of 1 mol/L Na_2_CO_3_. A sample blank (A_0_) was prepared identically but omitting enzyme (replaced with buffer), and a positive control (A_1_) contained enzyme and buffer in place of sample, while a background control (A_2_) contained buffer only (no sample, no enzyme). Absorbance was read at 405 nm on a microplate reader. Percent inhibition was calculated as: Inhibition = [1-(A-A0)/(A1-A2)] * 100%, where A is the absorbance of the sample plus enzyme reaction.

### 2.6 α-Amylase inhibition assay

α-Amylase activity was assayed by measuring reducing sugars released from soluble starch using the 3,5-dinitrosalicylic acid (DNS) method. In each 2 mL tube, 80 µL of test sample (100, 10, 1, 0.1, or 0.01 mg/mL) was combined with 80 µL of phosphate-buffered saline (PBS), then 80 µL of α-amylase (1 U/mL) was added. After 10 min pre-incubation at 37°C, 80 µL of 1% (w/v) soluble starch solution was introduced and the mixture incubated for an additional 10 min at 37°C. The reaction was terminated by adding 0.4 mL DNS reagent, followed by boiling in a water bath for 5 min. Tubes were then cooled under running tap water and absorbance measured at 540 nm. Controls were prepared as follows: sample blank (A_0_) with sample plus starch but no enzyme, positive control (A_1_) with enzyme and starch but no sample, and background control (A_2_) with starch alone (no sample, no enzyme). Inhibition was calculated using the same formula as above: Inhibition = [1-(A-A0)/(A1-A2)]* 100%, where A is the absorbance of the sample plus enzyme reaction.

### 2.7 Statistical analysis

Statistical analyses were performed using IBM SPSS Statistics (version 25.0). Continuous variables are presented as the mean ± standard deviation. Prior to group comparisons, datasets were assessed for normal distribution and homogeneity of variances. When comparing three or more groups, the one-way analysis of variance (ANOVA) was applied, depending on the experimental design. Following ANOVA, pairwise *post hoc* comparisons were carried out using the least significant difference (LSD) test or Tukey’s honestly significant difference procedure. A p-value less than 0.05 was considered indicative of statistical significance, while p < 0.01 denoted highly significant differences.

## 3 Results

### 3.1 Realgar and cinnabar effectively lowered blood glucose levels in STZ-induced diabetic mice

**Figure 1:**
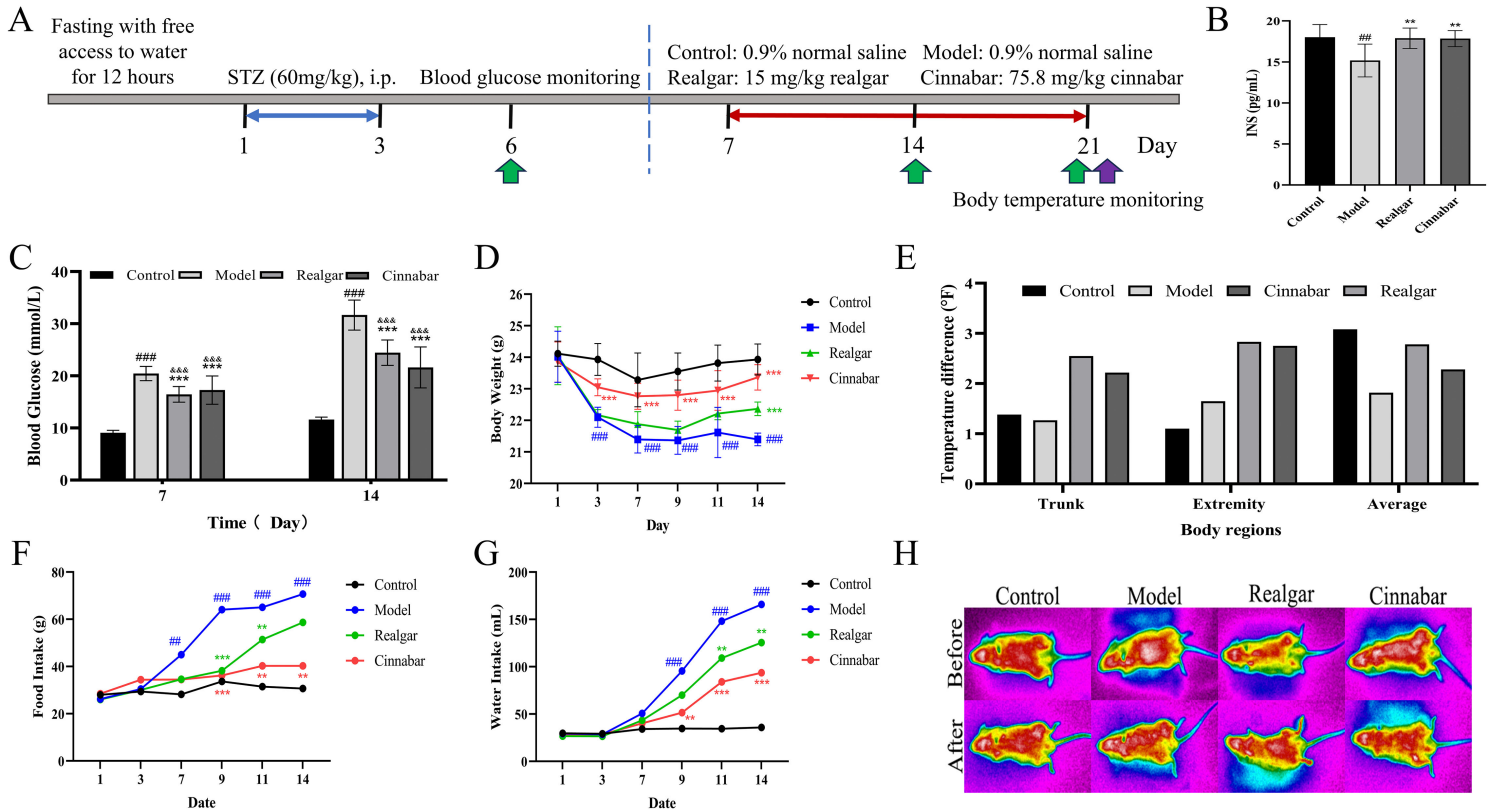
The effect of realgar and cinnabar on glucose metabolism in diabetic mice. **(A)** Experimental flowchart; **(B)** Insulin level; **(C)** Blood glucose profile; **(D)** Body weight; **(E)** Temperature difference; **(F)** Food intake; **(G)** Water intake; **(H)** Infrared thermogram. Compared to the control group, ###p < 0.01, &&&p < 0.01; compared to the model group, **p<0.05, ***p<0.01.

**Figure 1A** depicts the experimental workflow used to investigate the effects of realgar and cinnabar on glucose metabolism in STZ-induced diabetic mice. Random blood glucose was measured on days 7 and 14 (**Figure 1C**). Compared with the Control group (9.07 ± 0.48 mmol/L; 11.63 ± 0.45 mmol/L), the Model group exhibited significant hyperglycemia (20.45 ± 1.38 mmol/L; 31.67 ± 2.89 mmol/L; p < 0.01). Treatment with realgar and cinnabar markedly attenuated this STZ-induced rise in blood glucose at both time points (Realgar: 16.45 ± 1.51 mmol/L, 24.45 ± 2.44 mmol/L; Cinnabar: 17.27 ± 2.72 mmol/L, 21.62 ± 3.92 mmol/L; p < 0.01), indicating potent antihyperglycemic effects. Insulin assays revealed that, compared with the model group (15.18 ± 2.00 pg/mL), treatment with realgar (17.89 ± 1.25 pg/mL) or cinnabar (17.84 ± 0.98 pg/mL) elevated insulin levels, bringing them close to those observed in the control group (18.02 ± 1.54 pg/mL) (**Figure 1B**). Concomitantly, body weight, food intake, and water consumption were monitored throughout the 14-day treatment period (**Figures 1D, F, G**). Following STZ administration, Model mice experienced rapid weight loss, stabilizing below 22 g, and developed pronounced polyphagia and polydipsia, with cumulative food intake reaching 70.7 g and water intake 165.9 mL by day 14. Both realgar and cinnabar treatments significantly ameliorated these diabetic symptoms (p < 0.05), with cinnabar producing a more pronounced effect (p < 0.01). Body temperature was assessed before and after the 14-day treatment period (**Figure 1E**). The Model group showed minimal temperature change, mice receiving realgar or cinnabar exhibited modest increases of approximately 2–3°F in both trunk and extremity temperatures relative to baseline, although these differences did not reach statistical significance (p > 0.05). Representative infrared thermograms (**Figure 1H**) visually demonstrate the shifts in surface heat distribution before and after treatment.

### 3.2 Realgar and cinnabar significantly modulated glucose metabolism in normoglycemic mice

**Figure 2:**
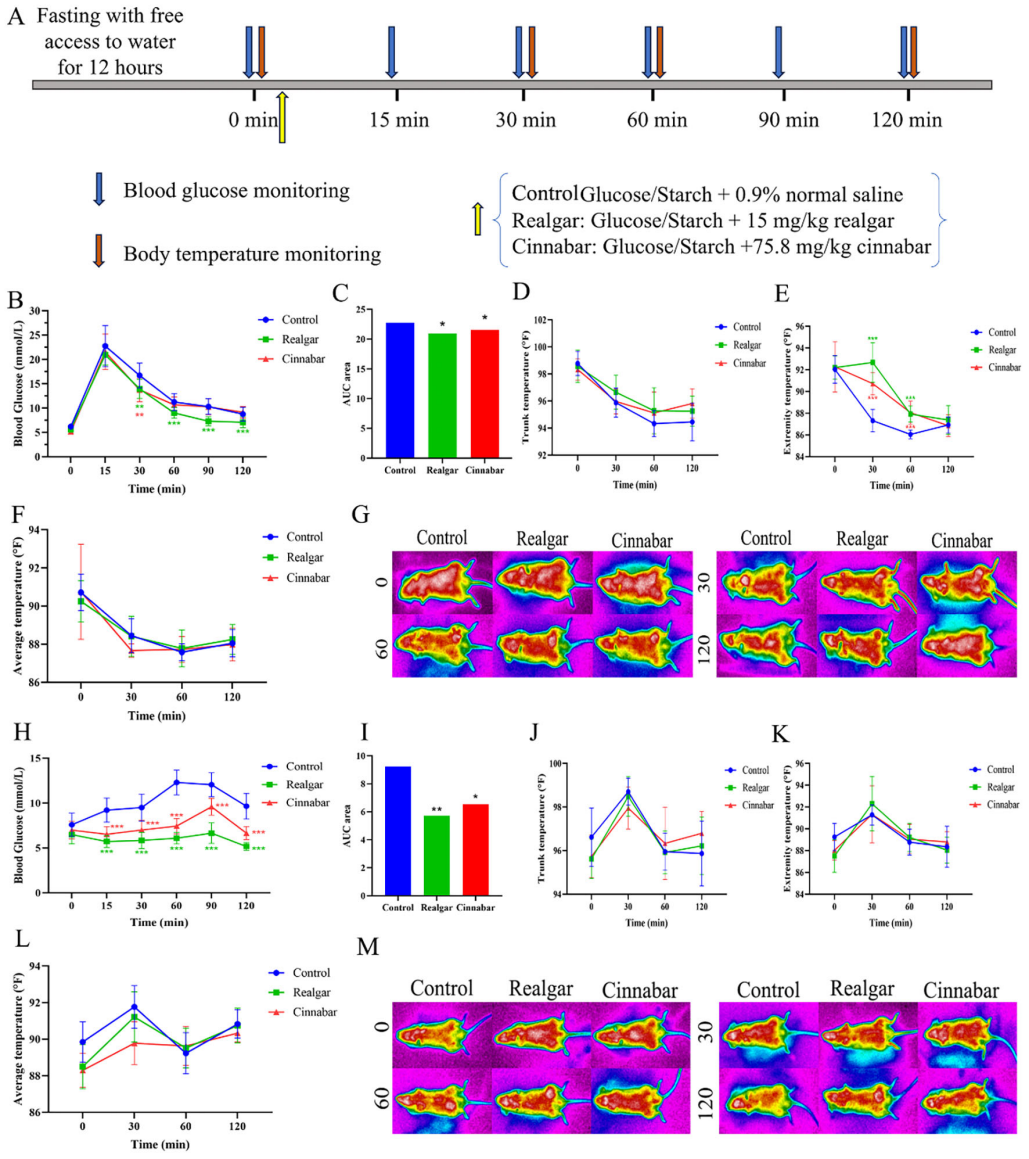
Effects of realgar and cinnabar on glycemic and thermoregulatory responses to glucose and starch challenges. **(A)** Experimental flowchart; **(B)** Blood glucose profile following oral glucose load. **(C)** AUC of blood glucose after glucose load. **(D)** Trunk temperature dynamics after glucose administration. **(E)** Extremity temperature changes after glucose administration. **(F)** Mean body temperature post-glucose gavage. **(G)** Representative infrared thermograms of mice after glucose loading. **(H)** Blood glucose profile following oral starch load. **(I)** AUC of blood glucose after starch load. **(J)** Trunk temperature dynamics after starch administration. **(K)** Extremity temperature changes after starch administration. **(L)** Mean body temperature post-starch gavage. **(M)** Representative infrared thermograms of mice after starch loading. Compared to the Control group, **p<0.05, ***p<0.01.

The effects of realgar and cinnabar on glycemic excursions and thermoregulatory responses in mice, following oral loads of either the monosaccharide glucose or the polysaccharide starch, are illustrated in **Figure 2**. After administering glucose according to the experimental protocol (**Figure 2A**), Control-treated mice exhibited a rapid rise in blood glucose, peaking at 15 minutes (22.76 ± 4.23 mmol/L), and then declining sharply from 30 minutes onward. Compared with the Control group (16.71 ± 2.53, 11.25 ± 1.76, 10.33 ± 1.61, and 8.75 ± 1.44 mmol/L at 30, 60, 90, and 120 minutes, respectively), mice treated with realgar showed significantly lower glucose levels at 30 min (13.96 ± 1.94 mmol/L, p < 0.05), 60 min (9.00 ± 1.08 mmol/L, p < 0.01), 90 min (7.31 ± 1.14 mmol/L, p < 0.01), and 120 min (7.10 ± 1.14 mmol/L, p < 0.01). Cinnabar-treated animals also exhibited a significant reduction at 30 minutes (13.75 ± 2.43 mmol/L, p < 0.05). By 120 minutes, blood glucose in all groups had returned to baseline (**Figure 2B**). Thermoregulatory monitoring revealed that, although core temperatures remained similar across groups (**Figure 2C**), extremity temperatures were significantly higher in the treated groups between 30 and 60 minutes (p < 0.01; **Figure 2D**). Representative infrared thermograms (**Figure 2F**) visually confirm the relative preservation of heat in the torso and limbs of treated mice.

In contrast, following an equivalent starch load, the Control group’s blood glucose rose more gradually, peaking at approximately 60 minutes (12.3 ± 1.38 mmol/L) and declining slowly thereafter. Both realgar and cinnabar groups reached lower peak levels at around 90 minutes (6.66 ± 1.14 and 9.60 ± 0.94 mmol/L, respectively) and maintained significantly reduced blood glucose concentrations from 30 to 120 minutes compared with controls (p < 0.01; **Figure 2G**). Thermally, starch challenge induced only mild and statistically nonsignificant reductions in trunk, extremity, and mean body temperatures across all groups (**Figures 2H, J**, p > 0.05), likely due to the slower enzymatic conversion of starch to glucose, which disperses heat production over a longer period. Infrared imaging (**Figure 2K**) showed broadly similar surface heat patterns across groups, with only minor localized differences.

### 3.3 Realgar and cinnabar abolish the hypoglycemic effect of intraperitoneally administered glucose but inhibit enzymatic activity

**Figure 3:**
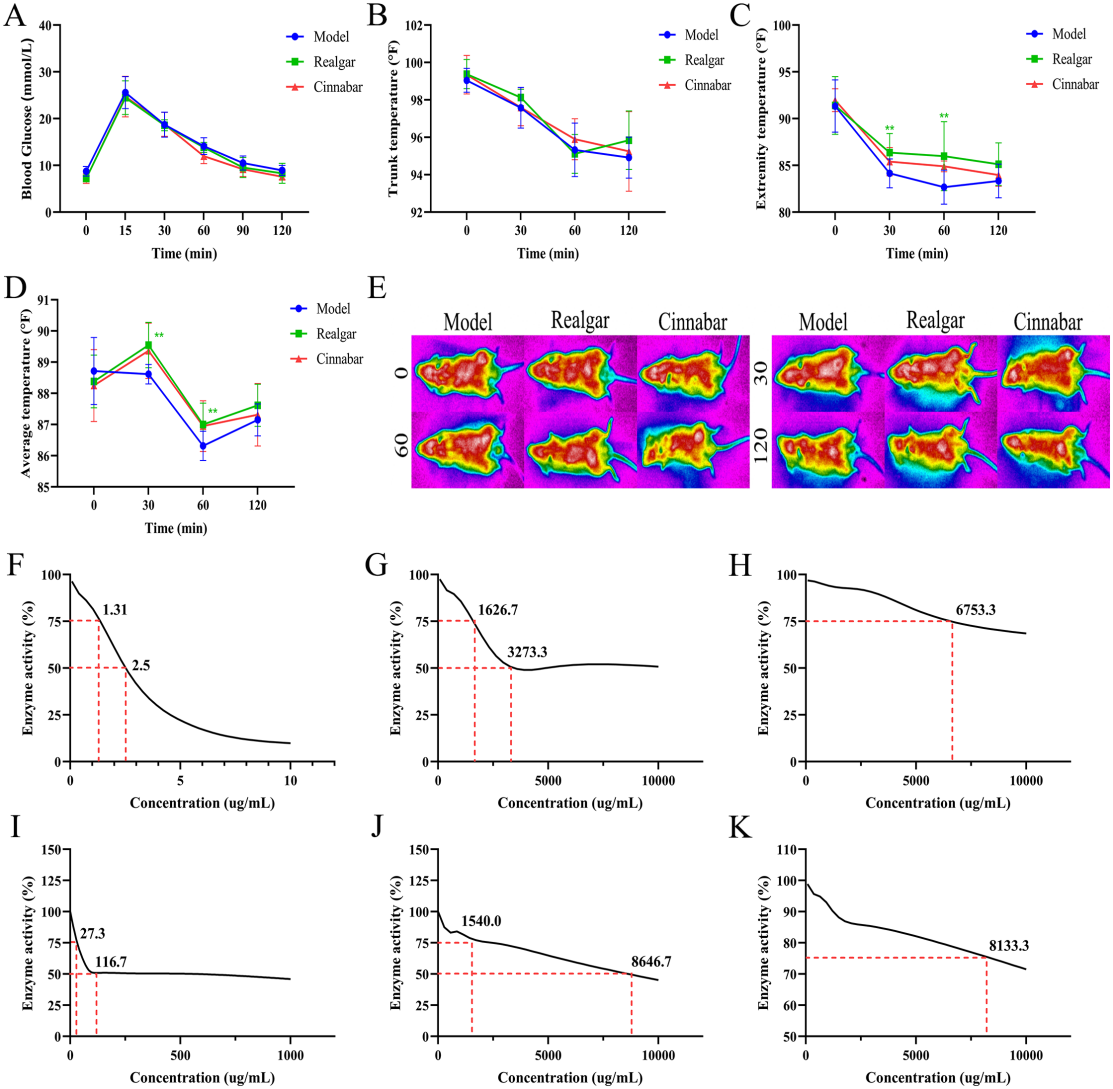
Realgar and cinnabar abolish the hypoglycemic effect of intraperitoneally administered glucose but inhibit enzymatic activity. **(A)** Blood glucose profile; **(B)** Trunk temperature; **(C)** Extremity temperature; **(D)** Whole-body mean temperature; **(E)** Infrared thermogram; **(F-H)** Enzymatic activity alterations of α-Glucosidase by Acarbose **(F)**, Realgar **(G)**, and Cinnabar **(H)**; **(I-K)** Enzymatic activity alterations of α -amylase by Acarbose **(I)**, Realgar **(J)**, and Cinnabar **(K)**. Compared to the Control group, **p<0.05, ***p<0.01.

Based on the findings above, we further investigated the role of realgar and cinnabar in glucose metabolism by assessing their effects on the physiological responses induced by intraperitoneal glucose administration, as well as their influence on key digestive enzyme activities. As shown in **Figure 3A**, blood glucose levels rose rapidly after glucose injection and peaked at 30 minutes before gradually declining. There were no significant differences in blood glucose levels between the realgar, cinnabar, and Control groups at any time point (p > 0.05), suggesting that the hypoglycemic effects of realgar and cinnabar were abolished under this administration route. Regarding thermoregulatory responses, **Figures 3B–D** indicate a time-dependent decrease in trunk, extremity, and whole-body average temperatures across all groups. However, at 30 and 60 minutes post-injection, both the realgar and cinnabar groups exhibited significantly higher extremity (**Figure 3C**) and average body temperatures (**Figure 3D**) compared to the Control group (p < 0.05). These observations were further supported by infrared thermographic imaging (**Figure 3E**), which revealed a broader distribution of high-temperature regions on the body surface in the realgar and cinnabar groups at 30 and 60 minutes.

The disappearance of the hypoglycemic effect of realgar and cinnabar following intraperitoneal glucose administration may suggest that their glucose-lowering actions, observed under oral glucose load, are primarily mediated via gastrointestinal mechanisms. To investigate this hypothesis, we examined the inhibitory effects of realgar and cinnabar on key digestive enzymes involved in carbohydrate breakdown, specifically α-glucosidase and α-amylase. As shown in **Figures 3F–H**, acarbose (positive control), realgar, and cinnabar all inhibited α-glucosidase activity in a dose-dependent manner, with IC_75_ values of 1.31, 1626.7, and 6753.3 µg/mL, respectively. Acarbose exhibited the strongest inhibition, while realgar demonstrated more potent enzyme inhibition than cinnabar. Similarly, **Figures 3I–K** show that all three agents also suppressed α-amylase activity, with IC_75_ values of 27.3, 1540.0, and 8133.3 µg/mL for acarbose, realgar, and cinnabar, respectively. Once again, realgar exhibited stronger biological activity than cinnabar in inhibiting enzymatic function, indicating its potential role in modulating glucose metabolism via inhibition of carbohydrate-digesting enzymes.

### 3.4 The metabolic impact of realgar and cinnabar on protein

**Figure 4:**
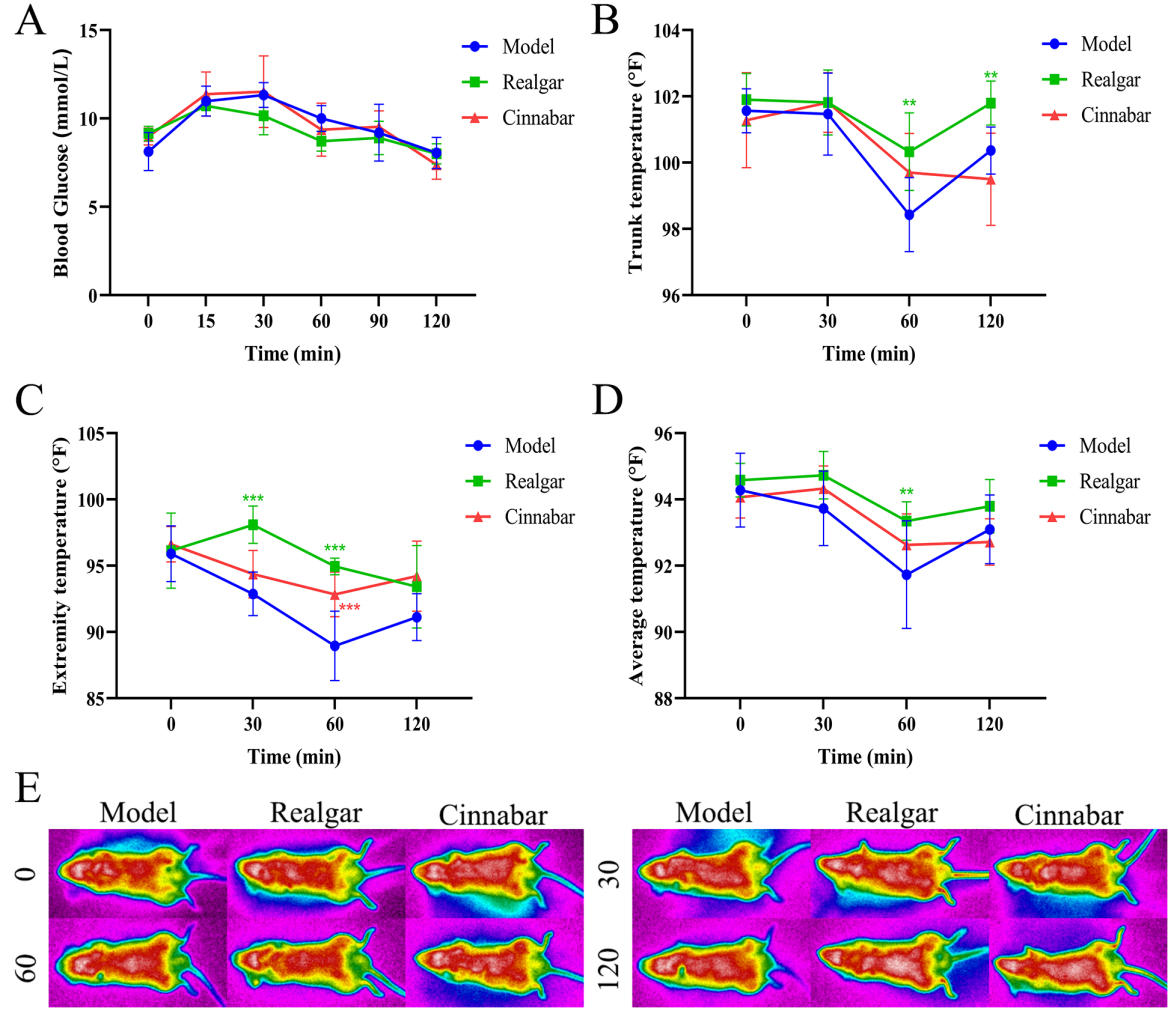
The metabolic impact of realgar and cinnabar on protein. **(A)** Blood glucose profile; **(B)** Trunk temperature; **(C)** Extremity temperature; **(D)** Whole-body mean temperature; **(E)** Infrared thermogram. Compared to the Control group, **p<0.05, ***p<0.01.

Following a protein challenge, mice in the Control, Realgar, and Cinnabar groups exhibited that elevated blood glucose and body temperature fluctuations responses. As shown in the glycemic profile (**Figure 4A**), blood glucose rose to peak levels at 15 and 30 minutes post-gavage, then declined steadily, returning to baseline by 120 minutes. Although glucose concentrations in the Realgar and Cinnabar groups were consistently lower than those in the Control group at each time point, these differences did not reach statistical significance (p > 0.05). Analysis of trunk, peripheral, and mean body temperatures (**Figures 4B–D**) demonstrated that a 12-hour fast followed by a protein-enriched load induced a transient hypothermic response, most pronounced at 60 minutes. At that time, trunk temperature in the Control group decreased to 98.43 ± 1.12°F, whereas Realgar- and Cinnabar-treated mice maintained higher trunk temperatures of 100.33 ± 1.17°F and 99.70 ± 1.19°F, respectively (Realgar *vs*. Control, p < 0.05). Extremity temperatures at 30 and 60 minutes were likewise elevated in the Realgar (98.10 ± 1.41°F) and Cinnabar (94.37 ± 1.79°F) groups compared with the Control group (82.88 ± 1.64°F; p < 0.01). These differences in regional temperatures resulted in a higher mean body temperature in the treatment groups relative to the Control group. Representative infrared thermographs (**Figure 4E**) provide a visual depiction of these temperatures across the torso and distal at each measured interval.

### 3.5 The metabolic impact of realgar and cinnabar on fat

**Figure 5:**
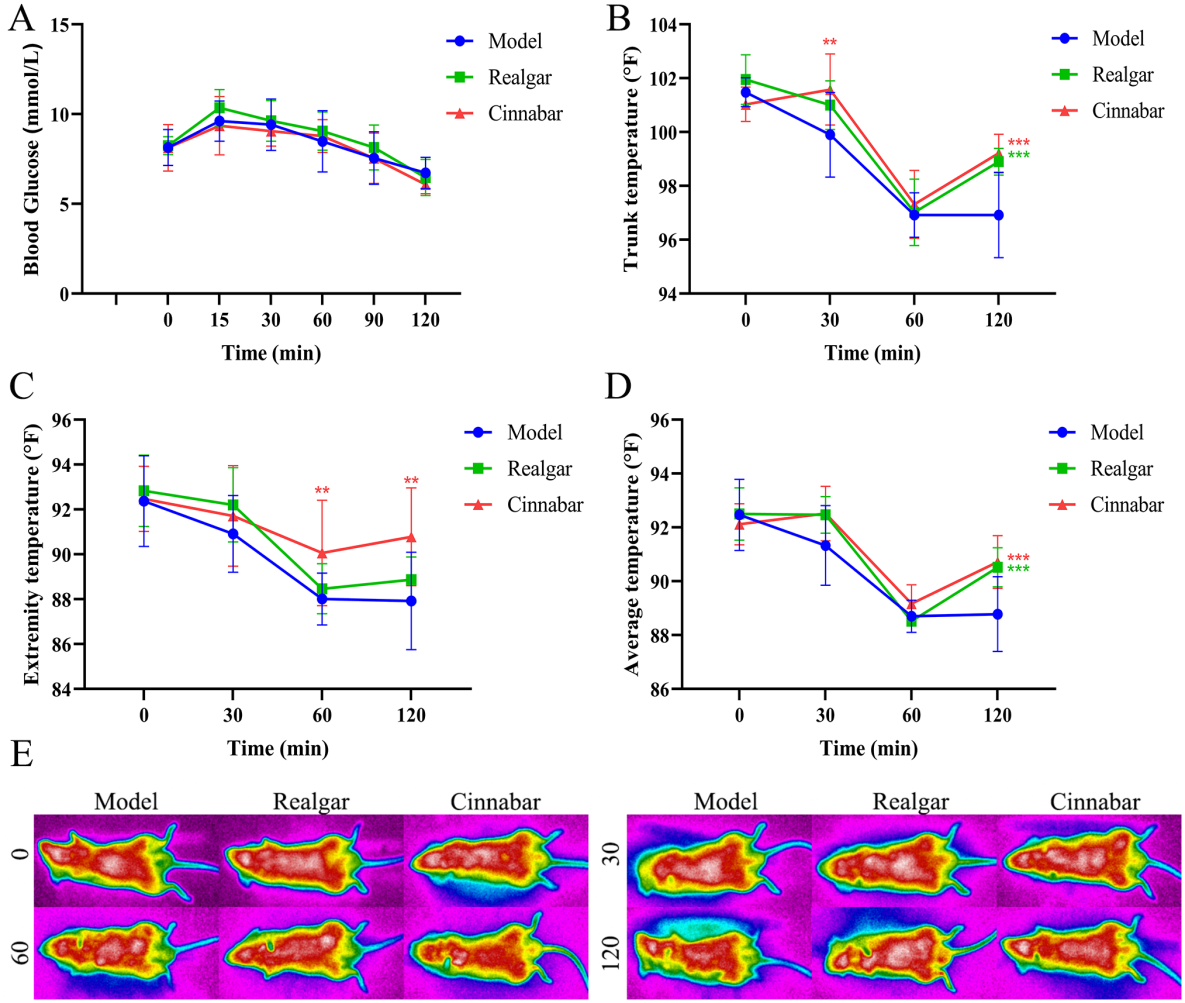
The metabolic impact of realgar and cinnabar on fat. **(A)** Blood glucose profile; **(B)** Trunk temperature; **(C)** Extremity temperature; **(D)** Whole-body mean temperature; **(E)** Infrared thermogram. Compared to the Control group, **p<0.05, ***p<0.01.

**Figure 6:**
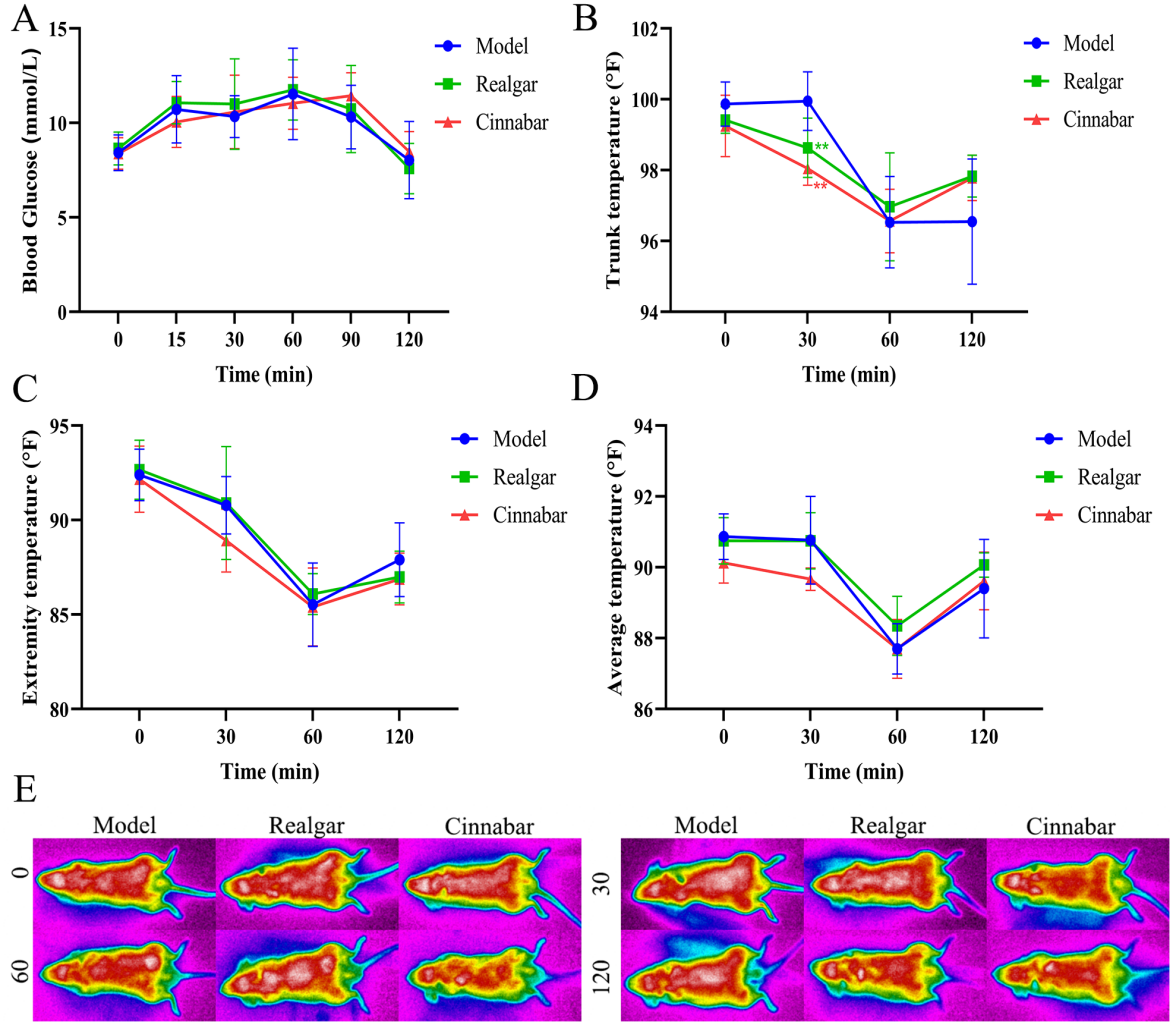
The metabolic impact of realgar and cinnabar on cellulose. **(A)** Blood glucose profile; **(B)** Trunk temperature; **(C)** Extremity temperature; **(D)** Whole-body mean temperature; **(E)** Infrared thermogram. Compared to the Control group, **p<0.05, ***p<0.01.

Following a high-quality lard challenge, all three groups—Control, Realgar, and Cinnabar—demonstrated the characteristic postprandial glycemic excursion of an initial rise followed by a gradual decline, however, the magnitude of blood glucose change was modest and did not differ significantly between groups at any time point (**Figure 5A**; p > 0.05). In contrast, oral lard loading produced pronounced effects on thermoregulation that were modulated by both mineral treatments (**Figures 6B–D**). At 120 minutes, trunk temperatures in the Realgar (98.90 ± 0.49°F) and Cinnabar (99.22 ± 0.71°F) groups were significantly higher than in the Control group (96.91 ± 1.58°F; p < 0.01) (**Figure 5B**). Extremity temperatures in the Cinnabar group were significantly elevated at both 60 minutes (90.07 ± 2.35°F) and 120 minutes (90.78 ± 2.18°F) compared with the Control group (88.01 ± 1.16°F and 87.92 ± 2.17°F, respectively; p < 0.05) (**Figure 5C**). Mean body temperature analyses further confirmed that, at 120 minutes, both Realgar- and Cinnabar-treated mice maintained significantly higher overall temperatures than controls (p < 0.01) (**Figure 5D**). Representative infrared thermographs (**Figure 5E**) visually depict these group‐specific alterations in surface thermal distribution across the torso and limbs, corroborating the quantitative temperature data.

### 3.6 The metabolic impact of realgar and cinnabar on cellulose

Following cellulose administration, mice in the Control, Realgar, and Cinnabar groups exhibited only mild glycemic fluctuations: blood glucose rose slightly between 15 and 30 minutes post-gavage, then stabilized and returned to baseline by 120 minutes, with no significant intergroup differences at any time point (p > 0.05). Concurrently, the effects of the cellulose load and mineral treatments on thermoregulation were assessed. At 30 minutes, trunk temperatures in the Realgar and Cinnabar groups were modestly lower than in the Control group (p < 0.05), but by 60 and 120 minutes, trunk temperatures converged across all groups, with no significant differences observed (**Figure 6B**). Extremity and mean body temperature profiles (**Figures 6C, D**) likewise showed only minor variations at 30 minutes, and otherwise time point with no statistically meaningful (p > 0.05). Overall, cellulose challenge induced relatively mild perturbations in both glucose homeostasis and thermoregulation, and neither Realgar nor Cinnabar altered the metabolic or thermal responses to dietary fiber.

## 4 Discussion

In mammals, glucose metabolism is a fundamental pathway for energy production and is tightly regulated by a neuroendocrine network and enzymatic cascades across key metabolic organs, including the liver, skeletal muscle, and adipose tissue (22). Disruptions in this finely tuned process impair systemic metabolic flexibility and can lead to glucose metabolism disorders, most notably diabetes mellitus (23). Both type 1 and type 2 diabetes are characterized by impaired glucose uptake and utilization (24), ultimately resulting in multi-organ dysfunction and chronic complications, typically manifesting as polydipsia, polyphagia, polyuria, and body weight loss.

In the present study, we demonstrated that the traditional mineral medicines Realgar and Cinnabar, both of which contain bioactive metal elements, exerted significant antihyperglycemic effects in STZ-induced diabetic mice. These effects were evidenced by reductions in random blood glucose levels, increased serum insulin concentrations, and alleviation of hallmark diabetic symptoms including weight loss, excessive drinking, and excessive eating. These findings suggest that realgar and cinnabar exert ​hypoglycemic effects​ *in vivo*, mediated not only by the ​restoration of pancreatic β-cell function, but also potentially through the ​modulation of peripheral tissue glucose utilization​ or other ​energy metabolism pathways​. Furthermore, thermal imaging and core temperature monitoring revealed that both mineral agents modestly elevated average and tail temperatures in diabetic mice, hinting at possible improvements in energy metabolism or peripheral circulation. Although these thermogenic effects did not reach statistical significance, they offer intriguing leads for further investigation.

Moreover, previous studies have highlighted that disturbances in glucose metabolism often precede the clinical onset of diabetes, suggesting that interventions during the prediabetic stage may offer greater therapeutic potential (25, 26). In our study, oral glucose and starch tolerance tests in normoglycemic mice revealed that both Realgar and Cinnabar effectively suppressed post-load glycemic excursions, with particularly pronounced effects observed in the starch-loading test. Supporting these *in vivo* findings, *in vitro* enzyme assays demonstrated that both minerals inhibited α-glucosidase and α-amylase activity, with Realgar exhibiting slightly stronger inhibition than Cinnabar. Since these enzymes are essential for the breakdown of dietary carbohydrates into absorbable monosaccharides in the intestine, the results suggest that the antihyperglycemic effects of Realgar and Cinnabar be mediated, at least in part, by attenuating intestinal carbohydrate digestion and absorption.

Notably, when glucose was administered via intraperitoneal injection, bypassing the gastrointestinal tract, the glucose-lowering effects of both realgar and cinnabar were abolished. This observation reinforces the hypothesis that their antihyperglycemic activity is dependent on gastrointestinal involvement, likely targeting enzymatic digestion within the intestinal lumen. Recent studies have highlighted the role of the gut–pancreas axis and nutrient-sensing signals in systemic glucose regulation, with gut-derived hormones such as GLP-1 playing pivotal roles in postprandial glucose control (27–29). Therefore, it is plausible that realgar and cinnabar may also modulate gut hormone secretion or gut microbiota composition, though this requires further investigation.

Additionally, by incorporating different nutrient loading models, including fat, protein, and cellulose, we evaluated the broader metabolic context of these agents. Under non-carbohydrate challenges, blood glucose levels remained relatively stable, and no statistically significant differences were observed between treated and control groups. This suggests that glucose metabolism was not strongly activated under these conditions and may have been supplanted by alternative energy pathways such as lipid oxidation or gluconeogenesis (30, 31). The lack of effect by realgar and cinnabar on glycemia in these settings implies that their actions are not mediated through broad metabolic regulation but are instead specifically triggered by carbohydrate intake, highlighting a distinct substrate-dependent mechanism.

In summary, this study is the first to validate the pharmacological role of realgar and cinnabar in the regulation of glucose metabolism, clearly demonstrating their ability to modulate hormonal responses and enzymatic activity under glucose load conditions in both diabetic and normoglycemic models. These findings not only expand our understanding of metallic mineral-based traditional medicines but also provide preliminary support for their potential application in managing metabolic diseases. Importantly, this work was designed as a proof-of-concept study, aiming to evaluate short-term glycometabolic responses and establish biological plausibility. This design allowed us to capture acute effects while minimizing systemic exposure risks associated with arsenic and mercury compounds. Nonetheless, several limitations should be acknowledged. First, our experiments focused on short-term interventions and acute metabolic load models, without evaluating long-term efficacy or safety. Although these minerals are poorly soluble, their arsenic and mercury content requires careful toxicological assessment in future studies, including chronic exposure and organ-specific effects. Second, mechanistically, our data are limited to systemic outcomes and enzyme activity. We did not investigate molecular or histological changes, such as gene expression or tissue alterations. Future studies should aim to evaluate chronic administration outcomes, and perform in-depth mechanistic analyses.

## 5 Conclusion

This study systematically demonstrates that realgar and cinnabar exert hypoglycemic effects in both STZ-induced diabetic mice and normoglycemic mice subjected to glucose and starch load, potentially through increasing insulin secretion and inhibiting key digestive enzymes involved in carbohydrate breakdown. These findings highlight the substrate-specific and potentially multifaceted metabolic actions of mineral-based agents, offering a novel perspective for developing targeted interventions in glucose and energy metabolism disorders.

## Data Availability

The datasets presented in this study can be found in online repositories. The names of the repository/repositories and accession number(s) can be found in the article/supplementary material.

## References

[1] Gancheva S Jelenik T Álvarez-Hernández E Roden M . Interorgan metabolic crosstalk in human insulin resistance. Physiol Rev. (2018) 98:1371–415. doi: 10.1152/physrev.00015.2017, PMID: 29767564

[2] O'Hearn M Lara-Castor L Cudhea F Miller V Reedy J Shi P . Incident type 2 diabetes attributable to suboptimal diet in 184 countries. Nat Med. (2023) 29:982–95. doi: 10.1038/s41591-023-02278-8, PMID: 37069363 PMC10115653

[3] IDF global clinical practice recommendations for managing type 2 diabetes 2025. Diabetes Res Clin Pract. (2025) 224:112238. doi: 10.1016/j.diabres.2025.112238, PMID: 40339700

[4] Genazzani AD Genazzani AR . Polycystic ovary syndrome as metabolic disease: new insights on insulin resistance. touchREV Endocrinol. (2023) 19:71–7. doi: 10.17925/EE.2023.19.1.71, PMID: 37313240 PMC10258623

[5] Yang FR Zhao YF Hu XW Liu ZK Yu XD Li CY . Nano-realgar suppresses lung cancer stem cell growth by repressing metabolic reprogramming. Gene. (2021) 788:145666. doi: 10.1016/j.gene.2021.145666, PMID: 33887368

[6] Keinan O Valentine JM Xiao H Mahata SK Reilly SM Abu-Odeh M . Glycogen metabolism links glucose homeostasis to thermogenesis in adipocytes. Nature. (2021) 599:296–301. doi: 10.1038/s41586-021-04019-8, PMID: 34707293 PMC9186421

[7] Kalra S Unnikrishnan AG Baruah MP Sahay R Bantwal G . Metabolic and energy imbalance in dysglycemia-based chronic disease. Diabetes Metab Syndr Obes. (2021) 14:165–84. doi: 10.2147/DMSO.S286888, PMID: 33488105 PMC7816219

[8] Hou Y Tan E Shi H Ren X Wan X Wu W . Mitochondrial oxidative damage reprograms lipid metabolism of renal tubular epithelial cells in the diabetic kidney. Cell Mol Life Sci. (2024) 81:23. doi: 10.1007/s00018-023-05078-y, PMID: 38200266 PMC10781825

[9] Hartsoe P Holguin F Chu HW . Mitochondrial dysfunction and metabolic reprogramming in obesity and asthma. Int J Mol Sci. (2024) 25:2944. doi: 10.3390/ijms25052944, PMID: 38474191 PMC10931700

[10] Smita RM Shuvo APR Raihan S Jahan R Simin FA Rahman A . The role of mineral deficiencies in insulin resistance and obesity. Curr Diabetes Rev. (2022) 18:e171121197987. doi: 10.2174/1573399818666211117104626, PMID: 34789132

[11] Dubey P Thakur V Chattopadhyay M . Role of minerals and trace elements in diabetes and insulin resistance. Nutrients. (2020) 12:1864. doi: 10.3390/nu12061864, PMID: 32585827 PMC7353202

[12] Guan H Xu Y Ma C Zhao D . Pharmacology, toxicology, and rational application of cinnabar, realgar, and their formulations. Evid Based Complement Alternat Med. (2022) 2022:6369150. doi: 10.1155/2022/6369150, PMID: 36204126 PMC9532072

[13] Tsoi B Wang S Gao C Luo Y Li W Yang D . Realgar and cinnabar are essential components contributing to neuroprotection of Angong Niuhuang Wan with no hepatorenal toxicity in transient ischemic brain injury. Toxicol Appl Pharmacol. (2019) 377:114613. doi: 10.1016/j.taap.2019.114613, PMID: 31207256

[14] Ran W Chen X Grant J Sharma S Mohammed KA Kumar A . Critical review on the effect and mechanism of realgar nanoparticles on lymphoma: state of the art on *in-vitro* biomedical studies. Recent Pat Nanotechnol. (2025) 19:581–91. doi: 10.2174/0118722105284287240621053904, PMID: 38982696

[15] Bai W Liu D Cheng Q Yang X Zhu L Qin L . Tetraarsenic tetrasulfide triggers ROS-induced apoptosis and ferroptosis in B-cell acute lymphoblastic leukaemia by targeting HK2. Transl Oncol. (2024) 40:101850. doi: 10.1016/j.tranon.2023.101850, PMID: 38043497 PMC10701457

[16] Yang R Chen F Xu H Guo Z Cao C Zhang H . Exploring the mechanism of realgar against esophageal cancer based on ferroptosis induced by ROS-ASK1-p38 MAPK signaling pathway. Evid Based Complement Alternat Med. (2022) 2022:3698772. doi: 10.1155/2022/3698772, PMID: 36133791 PMC9484897

[17] Song P Chen P Wang D Wu Z Gao Q Wang A . Realgar transforming solution displays anticancer potential against human hepatocellular carcinoma HepG2 cells by inducing ROS. Int J Oncol. (2017) 50:660–70. doi: 10.3892/ijo.2016.3831, PMID: 28035418

[18] Wang Y Wei Y Wu Y Zong Y Song Y Pu S . Multifunctional nano-realgar hydrogel for enhanced glioblastoma synergistic chemotherapy and radiotherapy: A new paradigm of an old drug. Int J Nanomedicine. (2023) 18:743–63. doi: 10.2147/IJN.S394377, PMID: 36820060 PMC9938708

[19] Yuan B Kikuchi H . Harnessing arsenic derivatives and natural agents for enhanced glioblastoma therapy. Cells. (2024) 13:2138. doi: 10.3390/cells13242138, PMID: 39768226 PMC11674460

[20] Committee for the Update of the Guide for the Care and Use of Laboratory Animals Institute for Laboratory Animal Research Division on Earth and Life Studies National Research Council . Guide for the Care and Use of Laboratory Animals (8th ed.). Washington, D.C.: National Academies Press (2011).

[21] King AJ . The use of animal models in diabetes research. Br J Pharmacol. (2012) 166:877–94. doi: 10.1111/j.1476-5381.2012.01911.x, PMID: 22352879 PMC3417415

[22] Roden M Shulman GI . The integrative biology of type 2 diabetes. Nature. (2019) 576:51–60. doi: 10.1038/s41586-019-1797-8, PMID: 31802013

[23] Darcey VL Guo J Chi M Chung ST Courville AB Gallagher I . Brain dopamine responses to ultra-processed milkshakes are highly variable and not significantly related to adiposity in humans. medRxiv. (2024) 37:616–28.e5. doi: 10.1016/j.cmet.2025.02.002, PMID: 40043691

[24] Laker RC Egolf S Will S Lantier L McGuinness OP Brown C . GLP-1R/GCGR dual agonism dissipates hepatic steatosis to restore insulin sensitivity and rescue pancreatic β-cell function in obese male mice. Nat Commun. (2025) 16:4714. doi: 10.1038/s41467-025-59773-4, PMID: 40399267 PMC12095689

[25] Tabák AG Herder C Rathmann W Brunner EJ Kivimäki M . Prediabetes: a high-risk state for diabetes development. Lancet. (2012) 379:2279–90. doi: 10.1016/S0140-6736(12)60283-9, PMID: 22683128 PMC3891203

[26] American Diabetes Association Professional Practice Committee . Diagnosis and classification of diabetes: standards of care in diabetes-2024. Diabetes Care. (2024) 47:S20–42. doi: 10.2337/dc24-S002, PMID: 38078589 PMC10725812

[27] Wang Q Lin H Shen C Zhang M Wang X Yuan M . Gut microbiota regulates postprandial GLP-1 response via ileal bile acid-TGR5 signaling. Gut Microbes. (2023) 15:2274124. doi: 10.1080/19490976.2023.2274124, PMID: 37942583 PMC10730136

[28] Duca FA Waise TMZ Peppler WT Lam TKT . The metabolic impact of small intestinal nutrient sensing. Nat Commun. (2021) 12:903. doi: 10.1038/s41467-021-21235-y, PMID: 33568676 PMC7876101

[29] Wachsmuth HR Weninger SN Duca FA . Role of the gut-brain axis in energy and glucose metabolism. Exp Mol Med. (2022) 54:377–92. doi: 10.1038/s12276-021-00677-w, PMID: 35474341 PMC9076644

[30] Franczyk MP Qi N Stromsdorfer KL Li C Yamaguchi S Itoh H . Importance of adipose tissue NAD+ Biology in regulating metabolic flexibility. Endocrinology. (2021) 162:bqab006. doi: 10.1210/endocr/bqab006, PMID: 33543238 PMC7853299

[31] Miyata Y Shimomura I . Metabolic flexibility and carnitine flux: The role of carnitine acyltransferase in glucose homeostasis. J Diabetes Investig. (2013) 4:247–9. doi: 10.1111/jdi.12064, PMID: 24843661 PMC4015659

